# Schwerpunkt: Krieg gegen die Ukraine

**DOI:** 10.1007/s41358-022-00339-x

**Published:** 2023-01-18

**Authors:** Manuela Glaab

**Affiliations:** Institut für Sozialwissenschaften, Abteilung Politikwissenschaft, Universität Koblenz-Landau, Kaufhausgasse 9, 76829 Landau in der Pfalz, Deutschland

Der am 24. Februar 2022 erfolgte Angriff Russlands auf die Ukraine markiert einen Epochenbruch. Darin sind sich die Beobachter einig, wenngleich die ganze Tragweite dieses Ereignisses erst in der Rückschau, mit größerem zeitlichem Abstand deutlich werden dürfte. Dass dieser Krieg in unmittelbarer Nachbarschaft der EU und das hierdurch verursachte Leid der Menschen in der Ukraine weitreichende (welt-)politische Konsequenzen haben würden, war die unausweichliche Erkenntnis. In seiner Rede vor dem Deutschen Bundestag, nur drei Tage nach Kriegsbeginn, brachte Bundeskanzler Scholz dies auf den Begriff der „Zeitenwende“, der die deutsche Öffentlichkeit auf „rapide Politikwechsel“ (Rüb 2014) einstimmte und seither die Debatte prägt. Eindringlicher noch formulierte Scholz es im Sommer 2022 vor einer deutsch-kanadischen Wirtschaftskonferenz (Brössler 2022): „Was wir gerade erleben, ist der perfekte Sturm“, sagte er, „eine Vielzahl sich überlappender und sich verstärkender Krisen und fundamentaler geopolitischer Verschiebungen.“

Der Krisendiskurs der vergangenen Jahre hat uns gelehrt, dass der allgegenwärtige Krisenbegriff sorgsam zu verwenden ist. Angesichts einer Serie sogenannter „transboundary crisis“, die geografische Grenzen und funktionale Schranken überschreiten, relativieren sich manch andere krisenhafte Erscheinungen. Auf die Euro- und Finanzkrise folgte bald die sogenannte „Flüchtlingskrise“ und schließlich die Covid 19-Pandemie mit ihren anhaltenden Folgeproblemen. Krisen weisen nach Boin et al. (2017) drei definitorische Merkmale auf: eine als akut wahrgenommene *Bedrohung* und die daraus resultierende *Dringlichkeit* zu handeln, bei gleichzeitig vorhandener *Unsicherheit*, was die Ursachen, den Charakter und die Konsequenzen derselben anbelangt. Charakteristisch für die Krisen der letzten Jahre ist zudem, dass sich der Anfangs- und Endpunkt kaum präzise bestimmen lässt, obwohl die einschlägige Literatur die Bedeutung der Rechenschaftslegung und des Lernens aus möglichen Fehlern des Krisenmanagements hervorhebt. Dies ist schon deshalb von Bedeutung, weil große Krisen das Potenzial haben, die Legitimationsgrundlagen und die Governance-Strukturen und -Prozesse demokratischer Systeme zu unterminieren. Krisen gelten gemeinhin als „Stunde der Exekutive“ (Wilharm 2022), da Verantwortliche in Regierung und Verwaltung zentrale Funktionen des Krisenmanagements wie auch der Krisenkommunikation übernehmen: „In a crisis, leaders are expected to reduce uncertainty and provide an authoritative account of what is going on, why it is happening, and what needs to be done“ (Boin et al. 2017, S. 17). Vor dem Hintergrund eines permissiven Konsenses, wonach bedrohliche Krisensituationen außergewöhnliche Maßnahmen rechtfertigen, können Spitzenakteure Gelegenheitsfenster politischer Führung nutzen, beispielsweise, indem sie institutionalisierte Entscheidungsregeln umgehen, um die als erforderlich erachteten Schritte kurzfristig durchzusetzen (Glaab 2022). Solange sie in der Lage sind, die Erwartungen zu erfüllen, mithin die Krise zu meistern und wieder Sicherheit herzustellen, ist zumindest die Output-Legitimation gewährleistet. Die Rückkehr zu den institutionalisierten Entscheidungsmodi und der Erhalt demokratischer Kontrolle sollten in konsolidierten Demokratien sodann verlässlich erfolgen. Wenn allerdings die Krise zum Normalfall wird, weil ein permanenter Krisenmodus in Politik und Gesellschaft vorherrscht, kann dies demokratische politische Systeme überfordern. Ebenso kann dies eintreten, wenn eine Krise sich derart verschärft, dass die Governance-Strukturen und -Prozesse insgesamt versagen.

Noch ehe die Ursachen und Folgewirkungen der erwähnten Krisenereignisse vollends behoben oder bewältigt sind, hat Putins Krieg gegen die Ukraine eine Krise weit größeren Ausmaßes ausgelöst, die Züge einer systemischen Herausforderung trägt. Die Umweltbedingungen und externen Anforderungen an das politische System haben sich mit dieser fundamentalen Regelverletzung derart verändert, dass auch der Systemerhalt im Innern gefährdet sein könnte. Damit befasst sich Hans Vorländer in seinem Beitrag, der aufzeigt, wie die „Gleichzeitigkeit von Krieg und Krisenbewältigung“ (ebd.) die Demokratie an ihre Belastungsgrenzen bringt. Dazu tragen nach Vorländer längerfristig zu beobachtende Entwicklungen bei; insbesondere die Autokratisierung ehemals liberaler Demokratien, allgemeiner aber auch ein „entkoppeltes Regierungshandeln“ (ebd.), das in plebiszitäre Reflexe, Populismus und Personenkult zu verfallen droht. Anlass zur Sorge gibt auch die Beobachtung, dass die intermediären, vermittelnden Institutionen der demokratischen Willensbildungs- und Entscheidungsprozesse in nicht wenigen Ländern schon länger erodieren und somit als politisch-kultureller Anker der Demokratie nur mehr bedingt Halt geben.

Optimistischer fällt die Einschätzung von Karl-Rudolf Korte aus, der in der Konstellation multipler Krisen zwar seinerseits einen fundamentalen „Gewissheitsschwund“ konstatiert. Im „transformativen Regieren“ erkennt er aber auch Möglichkeiten, zu neuen, politikfeldspezifischen Formen des Politikmanagements zu gelangen. Korte illustriert dies an jüngsten Entwicklungen der Berliner Ampelkoalition und sieht insbesondere den Bundeskanzler zu Führungshandeln aufgefordert. Wichtig ist dabei auch die These, dass der Krisenmodus des Regierens angesichts manifester Wohlstandsverluste nicht allein auf finanzielle Kompensation abstellen kann, sondern eine „inklusive Transformation, die auf Teilhabe und Teilnahme setzt“ (ebd.), kommunikativ vermitteln sollte.

Dass der Angriffskrieg Russlands gegen die Ukraine eine grundsätzliche und nachhaltige Regelverletzung in den internationalen Beziehungen darstellt, nehmen die Beiträge von Manuel Fröhlich und Stefan Schirm in den Blick. Fröhlich diskutiert die „Zeitenwende“ als Herausforderung für die deutsche Außenpolitik. Die russische Aggression in der Ukraine hat eine ganze Reihe von Grundkoordinaten der Bonner und Berliner Außenpolitik in Frage gestellt, wie sich insbesondere in der aktuellen Kontroverse um Wesen und Wert der deutschen „Ostpolitik“ zeigt. Fröhlich geht dem genauer auf den Grund, indem er die von unterschiedlichen theoretischen Standpunkten aus geführte Debatte um die normativen Grundlagen und den Gestaltungsanspruch deutscher Außenpolitik kritisch beleuchtet. Diese sei – ebenso wie der Blick auf die deutsche Außenpolitik der vergangenen Jahrzehnte – von einer Reihe problematischer Dichotomien geprägt, etwa von einem scheinbaren Gegensatz zwischen „wertgeleiteter“ Außenpolitik auf der einen und „realistischer“ Außenpolitik auf der anderen Seite (ebd.). Jenseits solcher Dichotomien stehe die deutsche Außenpolitik vor der Aufgabe, ihren Beitrag und ihr Selbstverständnis in der gegenwärtigen Krise der Weltordnung, konkreter des bestehenden Systems kollektiver Sicherheit, zu klären.

Russlands Überfall auf die Ukraine hat Stefan Schirm zufolge die Antagonismen zwischen dem „politischen Westen“, der von Nordamerika über Westeuropa bis nach Japan, Südkorea, Taiwan und Australien reicht, und dem „politischen Süden“, der von der BRICS-Gruppe angeführt wird, verschärft. Sichtbar werde dies in der Nichtbeteiligung jener Staaten an westlichen Militärhilfen für die Ukraine und den Sanktionen gegen Russland. Jedoch stehen den Institutionen der liberalen Weltordnung (LIO) schon seit Längerem andere internationale Organisationen gegenüber, die alternative gesellschafts- und wirtschaftspolitische Ordnungsvorstellungen favorisieren und die Prinzipien nationaler Autonomie und Nicht-Einmischung in interne Angelegenheiten vertreten. Derzeit seien die internationalen Beziehungen (noch) geprägt von Doppelmitgliedschaften, Multipolarität und Konkurrenz der Ordnungsvorstellungen, weshalb die gegenwärtige Phase ein Testfall für die Anziehungskraft der Modelle und die künftige Gestalt der Weltordnung sei (ebd.).

Die sogenannte Zeitenwende fordert Politik, Wirtschaft und Gesellschaft heraus, aber – darin stimmen die Autoren des aktuellen ZPol-Focus überein – auch die Politikwissenschaft steht vor Erklärungsbedarf. Bisherige Erkenntnisse müssen hinterfragt, teils radikal neue Perspektiven auf die Gegenstände des Fachs gerichtet werden. Herausgefordert scheint insbesondere die Teildisziplin der internationalen Beziehungen, aber auch die politische Systemforschung bis hin zur politischen Theorie sind gefragt. Die Debatte um die Zeitenwende ist im Gang und noch gibt es mehr Fragen als Antworten. Auch der Focus des vorliegenden Heftes liefert hierzu nur erste Impulse, dies jedoch in pointierter Form. Alle Beiträge wurden von Herausgebern der ZPol verfasst. Die Leserinnen und Leser der Zeitschrift sind dazu eingeladen, sich damit auseinanderzusetzen, Anregungen aufzugreifen und – selbstverständlich – eigene Schlussfolgerungen zu ziehen!

Prof. Dr. Manuela Glaab

Geschäftsführende Herausgeberin der Zeitschrift für Politikwissenschaft

PS: Der Leserschaft der ZPol sei ebenso wie den Autorinnen und Autoren hier noch mitgeteilt, dass die Geschäftsführende Herausgeberschaft samt Redaktion ab dem 1. Januar 2023 unter einem neuen institutionellen Dach firmieren: der neu gegründeten „Rheinland-Pfälzischen Technischen Universität Kaiserslautern-Landau“, kurz RPTU.
